# The Trend of Cefepime-Induced Neurotoxicity: A Systematic Review

**DOI:** 10.7759/cureus.40980

**Published:** 2023-06-26

**Authors:** Oluwafemi Ajibola, Taiwo O Aremu, Stephen O Dada, Olawale Ajibola, Kehinde O Adeyinka, Allicia Ajibola, Oluwatosin E Oluwole

**Affiliations:** 1 Pulmonary and Critical Care Medicine, Louisiana State University Health Sciences Center, New Orleans, USA; 2 Environmental Health Sciences, School of Public Health, University of Minnesota, Minneapolis, USA; 3 Pediatrics, University of Minnesota School of Medicine, Minneapolis, USA; 4 Pharmaceutical Care & Health Systems, College of Pharmacy, University of Minnesota, Minneapolis, USA; 5 Medicine, Windsor University School of Medicine, Cayon, KNA; 6 Medicine, American University of St. Vincent School of Medicine, Leeward Hwy, VCT; 7 Radiation Oncology, University College Hospital (UCH), Ibadan, NGA; 8 Nursing, Loyola University, New Orleans, USA; 9 Epidemiology & Community Health, School of Public Health, University of Minnesota, Minneapolis, USA

**Keywords:** antibiotic, nephrotoxic, glomerular filtration rate (gfr), encephalopathy, renal adjusted dose, creatinine clearance, fourth generation cephalosporin, renal impairment, cefepime-induced neurotoxicity, pseudomonas aeruginosa (p. aeruginosa)

## Abstract

There has been increased use of cefepime due to concerns about the nephrotoxic effects of the combined use of vancomycin and Zosyn. However, cefepime is associated with neurotoxicity.

We conducted a systematic review using online data to explore the trend of cefepime-induced neurotoxicity over the last 10 years. Forty-six articles met our inclusion criteria, including 73 cases of cefepime-induced neurotoxicity. We noticed a steady increase in the reports of cefepime-induced neurotoxicity, from one case in 2013 to 11 cases in 2022. Individuals aged 65 and older accounted for most cefepime-induced neurotoxicity cases (52%). The top three indications for cefepime administration included bone and joint infections (25%), urinary tract infections (22.7%), and pneumonia (22.7%). Most patients with renal impairment have never had a renal adjustment of their cefepime dosage (either 2 g 12 hours a day or 2 g eight hours a day). Most cases of cefepime-induced neurotoxicity occurred between days two and five (n=29, 71%), while most resolution occurred between days one and five (n=29, 85%). While cefepime continues to be a popularly used and effective antibiotic against gram-negative bacteria like *Pseudomonas aeruginosa*, its dosage needs to be adjusted in patients with renal impairment to avoid neurotoxicity.

## Introduction and background

Due to concerns about nephrotoxic effects from the combined use of vancomycin and Zosyn, there has been a shift and increased use of cefepime, a fourth-generation cephalosporin antibiotic, for its gram-negative coverage, especially *Pseudomonas*.

Cefepime is associated with neurotoxicity. The neurotoxicity associated with cefepime can manifest in various ways, including altered mental status, confusion, delirium, myoclonus (involuntary muscle jerks), seizures, and encephalopathy (brain dysfunction) [1], which can result in adverse health and economic implications [2]. The exact mechanism behind cefepime-induced neurotoxicity is not fully understood; however, it is believed to involve the drug's ability to cross the blood-brain barrier, which diminishes the inhibitory effect of the neurotransmitter ϒ-aminobutyric acid (GABA) [1]. The accumulation of cefepime in the brain that leads to the imbalance in neurotransmitter function is a precursor for observed neurotoxic symptoms [1].

Several risk factors have been identified for cefepime-induced neurotoxicity, including advanced age, underlying renal dysfunction, high doses of cefepime, and prolonged treatment duration [3,4]. Patients with pre-existing central nervous system disorders or impaired blood-brain barrier function may be more susceptible to neurotoxic effects [3].

The management protocol for cefepime-induced neurotoxicity recommends discontinuing cefepime and administering alternative antibiotics [1,5]. A delay in recognizing it can result in poor health outcomes, including prolonged hospital stays, intensive care unit (ICU) admissions, and death [2,6,7].

To better understand and characterize cefepime-induced neurotoxicity and recommend ways to mitigate it, this study aims to explore the trend of cefepime-induced neurotoxicity over the last 10 years using the online database.

## Review

Methods

On March 3, 2023, a methodical search was done on the PubMed website using the search word "cefepime neurotoxicity". The search was limited to publications in the English language in the last 10 years. Articles with case reports and case series were included. Other types of articles were excluded.

Two authors (OA and TOA) independently assessed the articles. Patient-specific information, including demographics, the dose of cefepime, indication for cefepime administration, symptoms at presentation, time of onset of symptoms after cefepime administration, concomitant medications, comorbidities, glomerular filtration rate (GFR), hemoglobin levels, lactic acid levels, blood pressure, imaging technique, time of symptom resolution, and ICU admission, was extracted using a prespecified data collection tool.

Results

**Figure 1:**
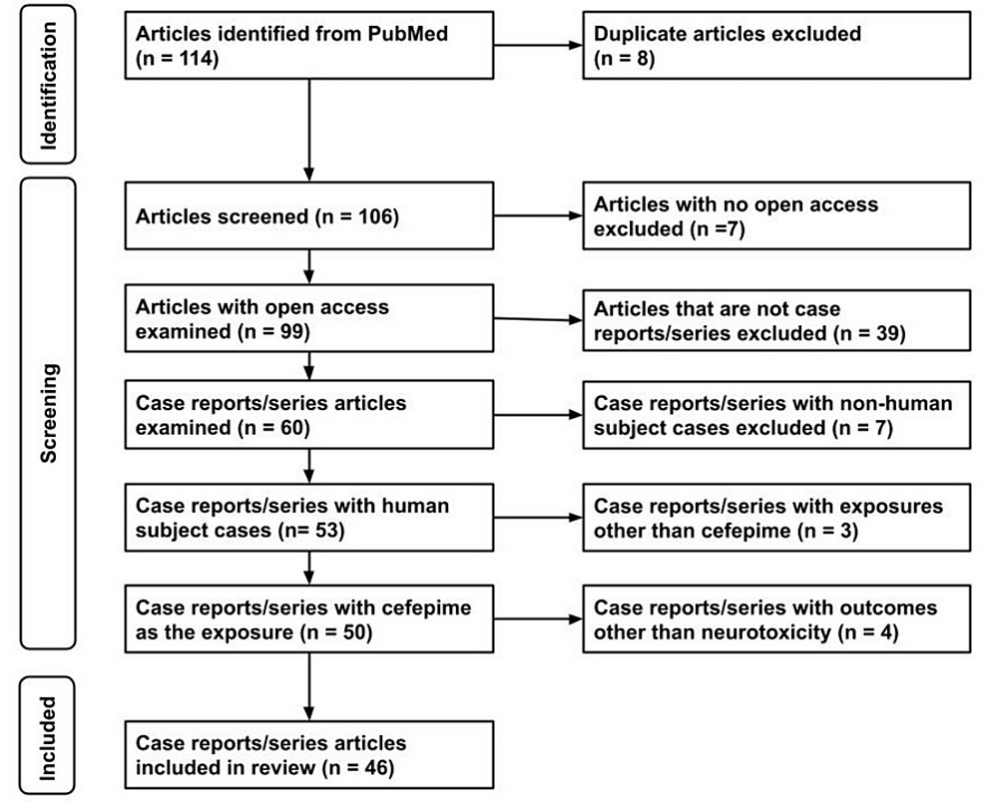
PRISMA flow chart PRISMA: Preferred Reporting Items for Systemic Reviews and Meta-Analyses; n = number of articles; PubMed = PubMed® database

Out of the 114 articles we identified, we excluded 68, including duplicates, non-open access articles, non-case reports or series, articles with non-human subject cases, articles with exposures other than cefepime, and articles with outcomes other than neurotoxicity. In essence, 46 articles met our inclusion criteria (Figure 1).

**Figure 2:**
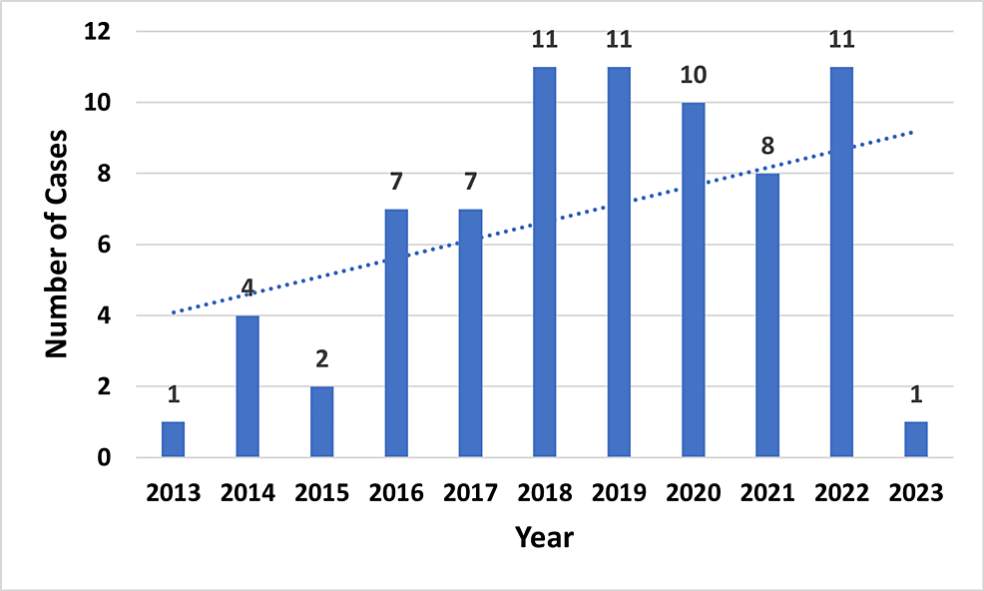
A chart showing the number of cases in the articles included per year considered (n=73)

We reviewed 46 articles and identified 73 cases of cefepime-induced neurotoxicity [8-53]. We noticed a steady increase in the reports of cefepime-induced neurotoxicity from one case in 2013 to 11 cases in 2022, depicting an increasing trend of cefepime administration through reported cases (Figure 2).

Among the 50 cases that had their age reported, individuals aged 65 and older accounted for the majority of cases (52% of cases) of cefepime-induced neurotoxicity (Table 1).

**Table 1:** Patients' distribution by parametric characteristics and categories CrCl: creatinine clearance

Parameters and Categories		N (% per category)
Age (n=50)	Birth – 1 month	0 (0)
	1 month – 1 year	0 (0)
	1 year – 12 years	2 (4.0)
	13 years – 17 years	1 (2.0)
	18 years – 64 years	21 (42.0)
	≥ 65 years	26 (52.0)
Indication for cefepime administration (n=44)	Urinary tract infection (UTI)	10 (22.7)
	Pneumonia (PNA)	10 (22.7)
	Soft tissue and skin infection	5 (11.4)
	Bone and joint infection	11 (25.0)
	CNS infection	0
	Bacteremia	4 (9.1)
	Biliary disease	2 (4.5)
	Neutropenic fever	2 (4.5)
CrCL (mL/min) (n= 31)	≥ 60	4 (12.9)
	30 – 59	9 (29.0)
	11 – 29	11 (35.5)
	< 11	6 (19.4)
	Hemodialysis	1 (3.2)

Forty-four reported cases of cefepime-induced neurotoxicity listed the indication for administering cefepime, with the top three including bone and joint infections (25%), urinary tract infections (22.7%), and pneumonia (22.7%). Thirty-one cases reported renal function (serum creatinine level or creatinine clearance). Out of the 31 cases that reported the serum creatinine level or the creatinine clearance, 87% had a creatinine clearance of less than 60 mL/min (Table 1). Out of the 31 cases that reported their renal function results, 18 reported the cefepime doses administered. 17 (94.4%) of the 18 cases that had their cefepime dose reported having renal impairment, defined as creatinine clearance less than 60 mL/min (Table 2).

**Table 2:** Reported renal function test results based on the recommended maintenance dose of cefepime

CrCL (mL/min)	Dosage of cefepime			
	2 g every 12 hours		2 g every 8 hours	
	Renal dose adjusted	Renal dose not adjusted	Renal dose adjusted	Renal dose not adjusted
30 – 60	0	6	3	3
11 – 29	0	5	2	3
< 11	0	5	0	5
Hemodialysis	0	1	0	1
Total	0 (0)	17 (100%)	5 (29%)	12 (71%)

The majority of patients with renal impairment did not receive cefepime after renal dose adjustment. Considering the recommended maintenance dose of 2 g every 12 hours, none of the patients were on renal adjusted dose, and when we considered the recommended maintenance dose of 2 g every eight hours, only 29% (n=5) of patients were on renal adjusted dose (Table 2).

Forty-one cases reported the onset of neurotoxicity after the initiation of cefepime. About 2.4% (n=1) of the cases had the earliest time of onset of 24 hours, while about 4.8% (n=2) of cases had the latest time of onset of four weeks. The majority of the cases of cefepime-induced neurotoxicity (n=29, 71%) occurred between days two and five (Table 3).

**Table 3:** Time of onset and resolution of neurotoxic symptoms following cefepime administration and discontinuation, respectively

Period (Day/Week)	Time of onset (n = 41)	Duration to the resolution of neurotoxicity after cefepime discontinuation (n = 34)
Day 1	1	4
Day 2	11	7
Day 3	4	9
Day 4	9	3
Day 5	5	6
Day 6	3	0
Day 7	2	2
Day 8	2	0
Day 9	0	1
Day 10	2	1
Week 4	2	1

Among the 34 cases that reported the duration of the resolution of the neurotoxicity after the discontinuation of the cefepime, the majority (n=29, 85%) had their symptoms resolved between days one and five.

Discussion

Antibiotics are among the most frequently used pharmaceuticals in inpatient and outpatient settings. While these antimicrobial agents are generally well tolerated, they are not without their associated side effects, both dose-dependent and idiosyncratic [4]. Neurotoxicity has been reported with first-generation cephalosporins such as cefazolin, second-generation cefuroxime, third-generation ceftazidime, and fourth-generation cefepime. It can range from encephalopathy to non-convulsive status epilepticus [27]. This is particularly true in cases of renal impairment, with some cases occurring in those with normal creatinine clearance [27]. In June 2012, the United States Food and Drug Administration (FDA) released a safety announcement reminding clinicians to adjust the dose of cefepime in patients with renal impairment because of the possibility of seizure activity as an adverse event [54]. Our findings showed that most reported cases had renal impairment from cefepime administration. This current study also showed that, at ages 18 and above, cefepime-induced neurotoxicity increased with increasing age. These findings agree with the anatomical and physiological changes in the kidneys with advancing age consequent upon normal organ senescence and some comorbidities such as atherosclerosis, diabetes mellitus, or hypertension that occur with greater frequency in older individuals [55]. With age, there is a decline in total nephron size and number, tubulointerstitial changes, glomerular basement membrane thickening, increased glomerulosclerosis, a decrease in glomerular filtration rate (GFR), and vascular changes; all these are further accelerated when exposed to toxins like drugs or when the patients are critically ill [56].

Cefepime clearance is largely renal and explained by the glomerular filtration rate, and at least 85% of cefepime is excreted unchanged in urine [50]. A retrospective study of critically ill patients on cefepime by Fugate et al. (2013) found that cefepime-induced neurotoxicity was significantly more frequent in patients without appropriate dose adjustments for renal function compared to those with dose reductions [57]. This evidence was also supported by the findings from our study, such that most of the cases that had renal impairment (CrCL ≤ 60 mL/min) were those that were not on renally adjusted doses of cefepime. When comparing cefepime to other broad-spectrum antibiotics we use in the hospital, cefepime has frequent dose modifications as renal function changes, which is very important in critically ill individuals or individuals who are at high risk for acute kidney injury [58]. Failure to recognize the decline in renal function and the need to modify the maintenance dose can lead to the neurotoxic effects of cefepime.

The typical time period for encephalopathy induced by cephalosporin use is a latency of one to 10 days following the start of medication and resolution in two to seven days following discontinuation [59-61]. We also found similar findings in our study, such that the majority of the neurotoxicity induced by cefepime occurred between days two and five, and the majority of the resolution occurred between days one and five after the discontinuation of the cefepime.

## Conclusions

Cefepime is an effective antibiotic with broad-spectrum coverage, particularly against gram-negative bacteria like *Pseudomonas aeruginosa*. However, it is important to administer cefepime with caution due to the potential neurotoxicity associated with its use. The incidence of cefepime-induced neurotoxicity has been increasing, likely due to the growing use of this antibiotic. Clinicians should be vigilant and informed about the rising trend of cefepime-induced neurotoxicity and take measures to mitigate its effects. Since cefepime is primarily eliminated through the kidneys, dose adjustment based on renal function is necessary, especially in critically ill patients or those at high risk for acute kidney injury. Close monitoring of kidney function is recommended, as cefepime dosage may need to be modified as renal function changes. Additionally, it is important to closely monitor the neurological status of patients receiving cefepime. Early recognition of cefepime-induced neurotoxicity can help minimize the duration of encephalopathy and reduce associated costs. Regular assessment of neurological function can aid in promptly identifying any signs or symptoms of neurotoxicity.
